# Racial and Ethnic Inequality in Survival Outcomes of Women With Triple Negative Breast Cancer

**DOI:** 10.7759/cureus.27120

**Published:** 2022-07-21

**Authors:** Sarah S Makhani, Antoun Bouz, Sarah Stavros, Isaac Zucker, Abigail Tercek, Katherine Chung-Bridges

**Affiliations:** 1 Department of Medical and Population Health Sciences Research, Herbert Wertheim College of Medicine, Miami, USA; 2 Department of Translational Medicine, Herbert Wertheim College of Medicine, Florida International University, Miami, USA; 3 Department of Medical and Population Health Sciences Research, Herbert Wertheim College of Medicine, Florida International University, Miami, USA; 4 Research, Health Choice Network, Miami, USA

**Keywords:** women’s health, disparities, race, survival, triple negative breast cancer

## Abstract

Purpose

Triple-negative breast cancer (TNBC) is the most lethal group of breast cancers. Socioeconomic factors may contribute to differences in survival rates. This study aims to identify racial/ethnic disparities in five-year survival rates among women affected by TNBC in the United States.

Methods

This retrospective study analyzed data from the 2010-2016 Surveillance, Epidemiology, and End Results Program database. Patients with a primary malignancy of triple-negative breast cancer were included in this study. Cancer-specific survival was measured at five years post-diagnosis. Cox regression models were used to calculate hazard ratios (HR) and corresponding 95% confidence intervals (CI).

Results

From 2010-2016, there were 26,963 women with a primary diagnosis of TNBC. After adjustment for age, insurance, marital status, stage, and surgery type, Hispanic women had the highest hazard of death when compared to White women (adjusted (adj) HR, 1.14, p<0.001). Further, non-Hispanic Black women also had a lower survival probability when compared to White women (adj HR, 1.06, p=0.002).

Conclusion

This study reveals that Hispanic women had the highest hazard of death when compared to White women. As TNBC is the most fatal breast cancer, future studies should investigate socioeconomic factors that may worsen prognosis of this disease.

## Introduction

Breast cancer, the most common malignancy and the second leading cause of cancer death in women, accounts for nearly one in four cancer cases [1, 2]. Although cancer mortality has significantly declined in the United States (US), significant racial and ethnic disparities persist in survival outcomes. While the incidence of breast cancer is higher in White women than Black women in the US, the mortality rate is higher in Black women [1, 3]. Biologically, unfavorable tumor receptor status, histological grade, and epigenetic mutations are more common in non-White patients [4]. In addition, studies have established the impact of sociodemographic factors on survival rates between different racial/ethnic groups [5, 6].

Triple-negative breast cancer (TNBC), the most lethal of breast cancers, is defined by a lack of expression of estrogen receptors (ER) and progesterone receptors (PR), without amplification of the human epidermal growth factor receptor (HER2/neu), which predisposes patients to fewer options for treatment [7, 8]. The disease burden of TNBC, which refers to morbidity, mortality, financial costs, and other factors, varies between races and ethnic groups, with Black women being the most affected group [9]. Studies hypothesize this disparity to be multifactorial, balancing possible hereditary susceptibility to mutant signaling pathways, access to quality medical care, comorbidity burden, and modifiable risk factors such as obesity and alcohol use [10].

Racial and ethnic disparities in breast cancer survival are already established [3,5,6]. However, breast cancer has a multitude of subtypes, receptor statuses, stages, and axillary lymph node involvement, which warrant further analysis [11]. Given the multifactorial nature of TNBC in Black women, identifying the key socioeconomic factors affecting survival is crucial in future trans-disciplinary prevention and treatment [12]. This study uses nationally representative data to investigate and add to the body of literature describing the impact and extent of racial disparities in survival among women with TNBC.

## Materials and methods

Study design

This retrospective cohort study used secondary data analysis from the Surveillance, Epidemiology, and End Results (SEER) database from 2010 to 2016. The SEER database collects and publishes data on cancer incidence and survival from population-based cancer registries in the United States. SEER*Stat software was utilized to extract data.

Study population

This study included adult women, 18 years of age or older, diagnosed with a primary malignancy of triple-negative breast cancer (TNBC). TNBC status was identified within the SEER database as ER-/PR-/HER2- [13]. Patients diagnosed at autopsy or death certificate were excluded from the study. Respondents with missing information on survival and race/ethnicity were also excluded. Since SEER is a de-identified, publicly available dataset, informed consent is not needed, and Florida International University’s Institutional Review Board deemed the study exempt from review.

The primary outcome of this study was five-year survival following diagnosis of TNBC, with cancer-specific death as the primary endpoint. This was calculated utilizing time from diagnosis to time of death due to TNBC. Race/ethnicity groups were categorized as “Non-Hispanic White”, “Non-Hispanic Black”, “Non-Hispanic Other”, and “Hispanic.” Demographic information was also collected as part of our secondary findings, including age at diagnosis (>18-39, 40-49, 50-59, 60-69, 70+), insurance status (private, Medicaid, uninsured, or not otherwise specified), and marital status (married or not married). Participants under the age of 40 were grouped together based on American Cancer Society mammography screening guidelines and the notion that breast cancer diagnosed before the age of 40 is more likely to be associated with aggressive disease and decreased survival [14]. Status as “not married” was defined by combining the SEER variables “single (never married)”, “divorced”, “widowed”, or “unmarried partner.” Tumor characteristics were identified including laterality (unilateral vs bilateral), grade (I-II or III-IV), and stage (I, II, III, or IV). Data on type of surgical treatment was classified as no surgery, partial mastectomy, subcutaneous mastectomy, total mastectomy, or modified/radical/extended mastectomy.

Statistical analysis

Five-year survival using the log-rank method was calculated using Stata/MP version 15.2 (StataCorp LLC, College Station, TX) with Kaplan-Meier survival curves. Baseline characteristics were reported for demographic and socioeconomic variables, and included percentages of each variable by race for both nominal and categorical variables. Further, a bivariate chi-squared analysis was used to identify possible confounders. Log-rank and Kaplan-Meier curves were used to compare survival between the racial groups. Unadjusted and adjusted Cox regression models at p<0.05 were used to calculate hazard ratios (HR), including corresponding 95% confidence intervals (CI). Variables controlled for included age, insurance, marital status, and surgery type.

## Results

Our study included a total of 26,963 women with a diagnosis of TNBC between 2010 and 2016. Higher proportions of Hispanic (16.8%), non-Hispanic Black (NHB) (9.0%), and non-Hispanic other (12.0%) women were diagnosed with TNBC before the age of 40, when compared to non-Hispanic White (NHW) (7.6%) (Table 1). Hispanic women had the highest proportion of uninsured status (4.3%) when compared to all other racial/ ethnic groups. Higher proportions of NHB (84.2%) and Hispanic (84.1%) women were diagnosed with high-grade tumors (grades III-IV) compared to NHW women (78.5%).

**Table 1:** Descriptive characteristics of women over 18 years of age diagnosed with triple-negative breast cancer in the Surveillance, Epidemiology, and End Results Program by Race/Ethnicity, 2010-2016. NOS: Not otherwise specified.

	Women, No. (%)				
Characteristics	White (n=16,127)	Black (n=5,312)	Other (n=2,086)	Hispanic (n=3,438)	p-value
Age, years					<0.001
18-39	1,221 (7.6)	478 (9.0)	251 (12.0)	576 (16.8)	
40-49	2,779 (17.2)	1,114 (21.0)	486 (23.3)	961 (28.0)	
50-59	4,118 (25.5)	1,680 (31.6)	521 (25.0)	915 (26.6)	
60-69	4,177 (25.9)	1,228 (23.1)	475 (22.8)	606 (17.6)	
70+	3,832 (23.8)	812 (15.3)	353 (16.9)	3,80 (11.1)	
Insurance					<0.001
Private	12,225 (80.0)	3,300 (63.2)	1,464 (71.2)	1,928 (56.9)	
Medicaid	1,351 (8.5)	1,033 (19.8)	320 (15.6)	973 (28.7)	
Uninsured	208 (1.3)	196 (3.8)	38 (1.9)	144 (4.3)	
Insurance NOS	2,100 (13.2)	695 (13.3)	233 (11.3)	345 (10.2)	
Marital Status					<0.001
Married	9,557 (62.4)	1,922 (38.4)	1,361 (68.8)	1,860 (57.4)	
Not Married	5,765 (37.6)	3,088 (61.6)	617 (31.2)	1,383 (42.7)	
Laterality					0.261
Unilateral	16,110 (99.9)	5,309 (99.9)	2,086 (100.0)	3,433 (99.9)	
Bilateral	17 (0.1)	3 (0.1)	0 (0.0)	5 (0.1)	
Grade					<0.001
Grade I-II	3,329 (21.5)	803 (15.8)	467 (23.3)	519 (15.9)	
Grade III-IV	12,129 (78.5)	4,267 (84.2)	1,537 (76.7)	2,752 (84.1)	
Stage					<0.001
Stage I	6,455 (40.9)	1,685 (32.5)	707 (35.0)	988 (30.0)	
Stage II	7,149 (45.3)	2,581 (49.8)	1,031 (51.0)	1,743 (53.0)	
Stage III	1,880 (11.9)	801 (15.4)	251 (12.4)	504 (15.3)	
Stage IV	297 (1.9)	121 (2.3)	34 (1.7)	56 (1.7)	
Surgery Type					<0.001
No Surgery	885 (5.5)	384 (7.3)	154 (7.4)	345 (10.1)	
Partial Mastectomy	8,331 (51.9)	2,859 (54.1)	906 (43.7)	1,522 (44.4)	
Subcutaneous Mastectomy	232 (1.4)	58 (1.1)	40 (1.93)	43 (1.3)	
Total Mastectomy	3,995 (24.9)	991 (18.7)	574 (27.7)	824 (24.1)	
Modified/Radical/Extended Mastectomy	2,615 (16.3)	998 (18.9)	401 (19.3)	692 (20.2)	

**Figure 1:**
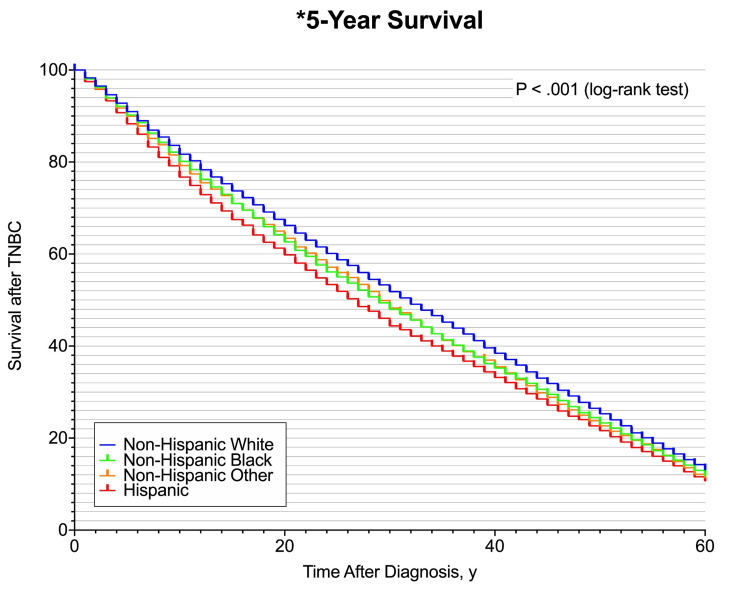
Five-year Kaplan-Meier Survival Curve TNBC: Triple-negative breast cancer

The five-year Kaplan-Meier survival estimates were significantly different among racial/ethnic groups, with Hispanic women having the lowest five-year survival estimate (10.7%, log-rank P < 0.001) compared to the other racial groups (NHW: 13.1%; NHB: 11.8%; Non-Hispanic other: 11.4%) (Figure 1).

After adjusting for age, insurance, marital status, stage, and surgery type, NHB (HR 1.07; 95% CI 1.03-1.11), non-Hispanic other, (HR 1.09; 95% CI 1.04-1.15), and Hispanic women (HR 1.15; 95% CI 1.10-1.20) had lower five-year survival rates compared to NHW women. Further, unmarried women demonstrated lower five-year survival when compared to married women (HR 1.04; 95% CI 1.00-1.06) (Table 2).

**Table 2:** Univariate and multivariate Cox regression analysis to determine differences in survival among racial groups.

	Five-Year Survival					
Characteristics	Crude Model			Multivariable		
	HR	(95%CI)	P-Value	HR	(95%CI)	P-Value
Race						
White	Ref			Ref		
Non-Hispanic Black	1.07	(1.04-1.11)	<0.001	1.06	(1.03-1.11)	0.002
Non-Hispanic Other	1.08	(1.03-1.14)	0.001	1.09	(1.04-1.15)	0.001
Hispanic	1.14	(1.10-1.19)	<0.001	1.14	(1.10-1.19)	<0.001
Age (years)						
18-39	Ref			Ref		
40-49	0.91	(0.87-0.96)		0.95	(0.90-1.00)	0.061
50-59	0.93	(0.89-0.98)	<0.001	1.00	(0.95-1.05)	0.967
60-69	0.95	(0.90-0.99)	0.031	1.03	(0.98-1.09)	0.161
70+	1.07	(1.02-1.13)	0.005	1.18	(1.12-1.24)	<0.001
Insurance						
Private Insurance	Ref		<0.001	Ref		
Medicaid	1.12	(1.08-1.17)	<0.001	1.05	(1.01-1.10)	0.013
Uninsured	1.05	(0.96-1.15)	0.262	1.00	(0.91-1.10)	0.993
Insurance NOS	0.97	(0.93-1.01)	0.155	0.95	(0.91-0.99)	0.018
Marital Status						
Married	Ref			Ref		
Not Married	1.08	(1.05-1.11)	<0.001	1.03	(1.00-1.06)	0.024
Laterality						
Unilateral	Ref			Ref		
Bilateral	1.02	(0.65-1.60)	0.940	*	*	*
Grade						
Grade I-II	Ref			Ref		
Grade III-IV	1.02	(0.99-1.06)	0.177	*	*	*
Stage						
Stage I	Ref			Ref		
Stage II	1.08	(1.52-1.11)	<0.001	1.08	(1.04-1.11)	<0.001
Stage III	1.22	(1.17-1.27)	<0.001	1.24	(1.19-1.30)	<0.001
Stage IV	2.25	(2.05-2.47)	<0.001	1.85	(1.67-2.05)	<0.001
Surgery Type						
No Surgery	Ref			Ref		
Partial Mastectomy	0.55	(0.52-0.58)	<0.001	0.60	(0.57-0.64)	<0.001
Subcutaneous Mastectomy	1.05	(0.93-1.17)	0.454	1.20	(1.06-1.35)	0.004
Total Mastectomy	0.60	(0.57-0.63)	<0.001	0.66	(0.62-0.70)	<0.001
Modified/Radical/ Extended Mastectomy	0.53	(0.50-0.52)	<0.001	0.54	(0.51-0.58)	<0.001

## Discussion

Our population-based study reports the impact of race on survival for TNBC patients using the SEER database. Current literature has identified racial disparities in breast cancer, suggesting that the relative risk of death is 71% higher for NHB women and 14% higher for Hispanic women, when compared to NHW women [11]. However, some literature suggests that race is not an independent prognostic indicator of survival for TNBC [15]. Ultimately, our analysis suggests that race/ethnicity is an independent predictor of five-year survival for the TNBC subtype. Compared to NHW women, there was lower five-year survival for Hispanic and NHB women in comparison to NHW women. When factoring in age, marital status, insurance, and surgical intervention in the adjusted analysis, racial disparities in survival outcomes persisted in our study.

There are a number of socioeconomic factors that may play a critical role in explaining the discrepancy between the results of our study and previous literature. Previous studies have described disparities in breast cancer outcomes due to varying socioeconomic status despite safety net insurance programs such as Medicare and Medicaid [16]. One study analyzed the role of neighborhood concentrated disadvantage index (CDI) on stage at diagnosis and stage-specific survival for TNBC patients. The goal of using CDI was to provide a robust proxy for both physical and social environments within the patient’s neighborhood. The results suggested that socioeconomic disadvantage contributes to racial disparities in both stages at diagnosis and stage-specific survival for TNBC patients [17]. These socioeconomic disparities may also contribute to the increased burden of disease seen in NHB and Hispanic patients compared to NHW patients [18, 19].

Insurance status, which is inextricably linked to socioeconomic status, has also been shown to impact both disease burden and survival. In a study in El Paso, Texas, women without access to insurance presented with a higher prevalence of TNBC [20]. Another study utilizing the SEER database suggests that racial disparities impacting prognosis were likely due to differences in medical insurance [21]. It is important to note that racial disparities in survival outcomes persisted in our study even after adjusting for insurance status, which suggests that differences in insurance status, and potentially disparate access to care, likely do not mediate the observed racial disparities. However, a secondary finding of note is that our study revealed poorer survival outcomes in patients with Medicaid when compared to private insurance.

Differences in receipt of guideline-concordant therapy may also influence racial disparities and cancer-specific survival. An analysis of the SEER database compared treatment differences between NHB and NHW, and reported that NHB women had lower odds of receiving surgery and chemotherapy when adjusting for sociodemographic and clinicopathologic factors [22]. Another study suggests that treatment and factors related to access to care account for differences in all-cause mortality between NHW and NHB women with TNBC, but not differences between Hispanic and NHW women [23]. Ultimately, socioeconomic status has implications on prognosis and survival through a variety of mechanisms, including environmental exposures and lifestyle factors that may modify risk, access to cancer care that is both timely and guideline-concordant, as well as access to primary care that allows for control of comorbidities.

In addition to social determinants, there may be genetic differences which partially explain perceived racial disparities. One analysis comparing NHB and NHW women reports that there may be racial differences in factors including somatic genomic mutations, population genetics, tumor heterogeneity, and increased expression of certain genes linked to breast cancer that lead to disparities in TNBC [24]. A review of several studies reports a number of ethnic-specific genetic biomarkers for TNBC [25]. Genes identified in Hispanic women in Northeast Mexico associated with an increased TNBC risk include HMGA1, FOXC1, and UGT8, among others [26]. An analysis of genetic markers in NHB women in the US identified markers that were indicators of poor prognosis, including CDKN1B, CLDN7, and DLC1 [27].

Classically, the literature has reported lower survival rates amongst NHB women with TNBC [12, 21, 22,28,29]. However, existing literature focuses on differences between NHB and NHW women, and there is a paucity of literature investigating the differences in survival and mortality for Hispanic women. This report adds to the body of literature comparing outcomes for NHB and Hispanic TNBC patients relative to NHW patients, it is important to note that there is not a clear consensus on the presence of differential survival outcomes. Interestingly, another SEER database analysis suggests that the hazard ratio for TNBC mortality is actually lower in Hispanic White women than NHW women [30].

Study limitations include absent individual-level socioeconomic factors such as income, high school education, and area of residence in the SEER database, as these factors may play a role in observed racial disparities in survival outcomes. In addition, the database also lacks detailed treatment history as well as risk factors for TNBC including family history, genetic predisposition, and weight, all of which could have played a role in disease prognosis.

## Conclusions

This study’s findings suggest that NHB and Hispanic women with TNBC have a lower five-year survival compared to NHW women. Targeting disparities in TNBC requires a multifaceted approach, incorporating the epigenetics, social determinants of health, and behavioral factors to determine the highest mortality predictors among racial and ethnic groups. As more approaches to treatment continue to unfold, the patterns of survival among racial groups may inform all-inclusive prevention, screening, and treatment recommendations.
